# Association of Mitochondrial DNA Polymerase γ Gene *POLG1* Polymorphisms with Parkinsonism in Chinese Populations

**DOI:** 10.1371/journal.pone.0050086

**Published:** 2012-12-10

**Authors:** Ya-xing Gui, Zhong-ping Xu, Wen Lv, Hong-mei Liu, Jin-Jia Zhao, Xing-Yue Hu

**Affiliations:** 1 Department of Neurology, Sir Run Run Shaw Hospital, Affiliated with School of Medicine, Zhejiang University, Hangzhou, Zhejiang, China; 2 Institute of Health Science, Shanghai Institutes for Biological Sciences, Chinese Academy of Sciences, Shanghai, China; 3 Department of Medicine, University of Chicago, Chicago, Illinois, United States of America; Oslo University Hospital, Norway

## Abstract

**Background:**

Mitochondrial DNA polymerase gamma (*POLG1*) mutations were associated with levodopa-responsive Parkinsonism. *POLG1* gene contains a number of common nonsynonymous SNPs and intronic regulatory SNPs which may have functional consequences. It is of great interest to discover polymorphisms variants associated with Parkinson's disease (PD), both in isolation and in combination with specific SNPs.

**Materials and Methods:**

We conducted a case-control study and genotyped twenty SNPs and poly-Q polymorphisms of *POLG1* gene including in 344 Chinese sporadic PD patients and 154 healthy controls. All the polymorphisms of *POLG1* we found in this study were sequenced by PCR products with dye terminator methods using an ABI-3100 sequencer. Hardy-Weinberg equilibrium and linkage disequilibrium (LD) for association between twenty *POLG1* SNPs and PD were calculated using the program Haploview.

**Principal Results:**

We provided evidence for strong association of four intronic SNPs of the *POLG1* gene (new report: c.2070-12T>A and rs2307439: c.2070-64G>A in intron 11, P = 0.00011, OR = 1.727; rs2302084: c.3105-11T>C and rs2246900: c.3105-36A>G in intron 19, P = 0.00031, OR = 1.648) with PD. However, we did not identify any significant association between ten exonic SNPs of *POLG1* and PD. Linkage disequilibrium analysis indicated that c.2070-12T>A and c.2070-64G>A could be parsed into one block as Haplotype 1 as well as c.3105-11T>C and c.3105-36A>G in Haplotype 2. In addition, case and control study on association of *POLG1* CAG repeat (poly-Q) alleles with PD showed a significant association (P = 0.03, OR = 2.16) of the non-10/11Q variants with PD. Although intronic SNPs associated with PD didn't influence *POLG1* mRNA alternative splicing, there was a strong association of c.2070-12T>A and c.2070-64G>A with decreased *POLG1* mRNA level and protein levels.

**Conclusions:**

Our findings indicate that *POLG1* may play a role in the pathogenesis of PD in Chinese populations.

## Introduction

Parkinson's disease (PD) is one of the most frequent neurodegenerative disorders caused by loss of dopaminergic neurons in the substantia nigra [1]. 5–10% of PD cases begin at onset of the ages between 20 and 50, which is classified as young onset [2]. The majority of PD patients are sporadic cases [2]. Approximately 5–10% of patients have genetic factors, yet the etiology of PD remains unclear [3], [4]. Mitochondrial dysfunction was implicated in the pathogenesis of sporadic, idiopathic PD [5], [6], [7]. Accumulation of somatic mitochondrial DNA (mtDNA) deletions was observed in the substantia nigra in PD and several gene products of familial Parkinsonism, e.g., mitochondrial DNA polymerase gamma gene (*POLG1*), had a connection to mitochondrial function [2], [5], [7].

Numerous evidences indicated that *POLG1* mutations cosegregated with levodopa-responsive Parkinsonism in some families, suggesting that *POLG1* could be responsible for some mendelian transmission of Parkinsonism [5], [6], [8], [9]. *POLG1* encodes part of the heterotrimeric mitochondrial DNA polymerase consisting of a catalytic subunit [5], [10], which is essential for mtDNA replication and repair due to its 5′ to 3′ polymerase activity and 3′ to 5′ exonuclease activity. *POLG1* mutations lead to uncorrect nucleotide incorporation in mtDNA, which resulted in dysfunction of the respiratory chain [11]. Homozygous knock-in mice expressing a polymerase deficiency mutant of *POLG1* shown an phenotype with increased amounts of point mutations and deleted mtDNA [12]. In some cases, mitochondrial DNA primary genetic abnormalities or secondary rearrangements due to *POLG1* gene mutation could directly cause Parkinsonism [5], [9]. Two missense mutations in *POLG1*, G737R and R853W, were reported in a family with early onset Parkinsonism [13]. Another W748S missense mutation was found in a Finnish patient with a mixed disease background of Parkinsonism, chronic progressive external ophthalmoplegia, and peripheral neuropathy [14]. *POLG1* contains an N-terminal polymorphic polyglutamine tract encoded by a (CAG)_n_ tract in exon 2 which may have functional consequences [6], [15]. Increased frequency of rare alleles of the *POLG1* CAG-repeat (poly-Q) was found in Finnish idiopathic apparently sporadic PD patients, but conflicting reports exist. Therefore, the gene for *POLG1* may represent an ideal candidate gene for Parkinsonism susceptibility. If susceptibility alleles are common in the general population and have rather small effects, association can be a more sensitive method than linkage to identify these loci. It is therefore of great interest to discover specific polymorphism variants of *POLG1* associated with PD.

Here we scrutinized the genetic polymorphisms in *POLG1* gene to evaluate it as potential candidate gene for PD genetic study. We performed extensive screening of *POLG1* gene by direct sequencing to detect polymorphisms, and statistical analysis to examine the genetic effects on PD in Chinese populations.

## Materials and Methods

### Sample collection and DNA/RNA preparations

A total of 498 study subjects were included in this study, comprising 344 patients with PD (175 males and 169 females) and 154 control subjects without evidence of PD (84 males and 70 females). We recruited all patients from the Department of Neurology from Sir Run Run Shaw hospital affiliated with Zhejiang University School of Medicine and received a standard neurological examination as well as a psychiatric interview. The PD patients with a mean age of 58.38±9.32 years (age at onset 54.22±8.52). The clinical diagnosis of PD was confirmed by the senior neurologist specializing in movement disorders according to the Consensus Statement of the Movement Disorders Society in 1998 [16]. The healthy control subjects (mean age of 59.57±10.86 years old) were free of neurological or psychiatric disorders. Gender and age proportion between cases and controls was statistically matched. All subjects were with Chinese Han ethnic background. Informed consent for participation in the study was obtained from either directly or from his or her guardian in all subjects and the work received approval from the institution ethics committee and conformed to the tenets of the Declaration of Helsinki. After informed consent was obtained from all participating individuals, blood samples were collected for genomic RNA/DNA extraction using the QIAamp Blood kit (Qiagen). To avoid genomic DNA contamination in RNAs, samples were treated with DNase I (Qiagen). All samples were tested by real-time PCR, showing no amplification after 40 cycles in cDNA preparation without RT.

Another group of 110 patients with sporadic early-onset Parkinsonism was studied. All the patients were from Hubei province, Central China. There were 62 male and 48 female sporadic patients: mean age at onset, 32.7±6.4 years. All patients underwent a standardized neurological examination by two movement disorder specialists. 110 PD patients were selected according to the following criteria: (1) at least two of the three cardinal motor signs (resting tremor, bradykinesia, rigidity); (2) excellent response following L-Dopa therapy; (3) an age at onset ≤40 years; (4) Absence of extensor plantar reflexes, ophthalmoplegia, early dementia, or early autonomic failure in the family members. A control group of 130 healthy individuals from the same geographic areas was obtained. All controls were free of symptoms suggestive of PD and with a negative family history of movement disorders. Informed consent was obtained before participation into our study.

### PCR and SNP screening by sequencing

To find common SNPs, we designed specific primer sets based on the *POLG1* sequences (NM_002693) in the GenBank DNA database to screen the exons and the flanking regions, including the promoter regions (1.5 kb) of these genes. The first-step SNP discovery screening panel included 15 affected and 10 unaffected individuals. PCR conditions were set as follows: 2 min initial denaturation at 95°C, 30 cycles of 30 s denaturation at 94°C, 30 s annealing at 53–60°C, 30 s extension at 72°C and 10 min final extension at 72°C. Before sequencing, amplification products were incubated with shrimp alkaline phosphatase (0.5 units; Amersham) and exonuclease I (1 units; Amersham) at 37°C for 30 min, followed by heat inactivation at 80°C for 15 min. The DNA samples containing extension products were analyzed using the sequencing kit ABI PRISM dye terminator cycle and the automatic sequencing system ABI 3100 (Applied Biosystems).

Twenty SNPs within the *POLG1* gene were found in twenty-five individuals, including ten exonic SNPs and ten intronic SNPs (Table 1). There is no other polymorphism with a frequency over 4% in all exons and flanking regions of *POLG1*. We further conducted a case-control study on these twenty SNPs of *POLG1* gene from the subjects, including 344 affected PD patients and 154 unaffected individuals, by PCR-direct sequencing using the ABI-3100 sequencer. The twenty-five samples in the SNP discovery panel were included in the case-control study and calculated in the final significance analysis. Specific primers were designed based on genomic sequences obtained from the GenBank DNA database.

**Table 1:** Overview of the 20 SNPs of the human *POLG1* gene that is genotyped in our study by direct sequencing.

Gene name	SNP Name^a^	Location	Polymorphism	AA Variation	Position*	dbSNP ID§	PCR Primer
*POLG1*	SNP89867197	Intron11	c.2070-64G>A	___	89867197	rs2307439	Forward 5′>cctgtacaggaagcactgtct<3′; Rerverse 5′>gagaacccaagccggcgcact<3′
*POLG1*	SNP89867154	Intron11	c.2070-21T>C	___	89867154	rs2072267	Forward 5′>cctgtacaggaagcactgtct<3′; Rerverse 5′>gagaacccaagccggcgcact<3′
*POLG1*	SNP89867145	Intron11	c.2070-12T>A	___	89867145	New report	Forward 5′>cctgtacaggaagcactgtct<3′; Rerverse 5′>gagaacccaagccggcgcact<3′
*POLG1*	SNP89866976	Intron10	c.2070+158G>A	___	89866976	New report	Forward 5′>acctgggatggcttccctctg<3′; Rerverse 5′>agcccatctcccagctactt< 3′
*POLG1*	SNP89866286	Intron13	c.2266-153C>T	___	89866286	rs3176202	Forward 5′>caatggaccttacaacgacgt<3′; Rerverse5′>caaaggggcttcccacatta<3′
*POLG1*	SNP89866954	Intron12	c.2157-211T>C	___	89866954	rs2072266	Forward 5′>acttagaagtggaggctgag<3′; Rerverse 5′>gttgtaaggtccattgccat<3′
*POLG1*	SNP89866804	Intron12	c.2157-61C>T	___	89866804	rs3176200	Forward 5′>acttagaagtggaggctgag<3′; Rerverse 5′>gttgtaaggtccattgccat<3′
*POLG1*	SNP89878574	Promoter	c.-830A>G	___	89878574	rs3176150	Forward 5′>agctccagccgcgctgtgg<3′; Rerverse 5′>ttcgctggcagccgcaactt<3′
*POLG1*	SNP89862341	Intron19	c.3105-11T>C	___	89862341	rs2302084	Forward 5′>gatttccctgcaggatctgcg<3′; Rerverse 5′>atgcagcagcccagcaccggg<3′
*POLG1*	SNP89862366	Intron19	c.3105-36A>G	___	89862366	rs2246900	Forward 5′>gatttccctgcaggatctgcg<3′; Rerverse 5′>atgcagcagcccagcaccggg<3′
*POLG1*	SNP89873445	Exon2	c.722C>T	P241L	89873445	rs56410699	Forward 5′>atcatctgtctcctttag<3′; Rerverse 5′>ctgaaacaaactattaagc<3′
*POLG1*	SNP89871763	Exon4	c.1174C>G	L392V	89871763	rs145289229	Forward 5′>tctgtatttggtgttgaag<3′; Rerverse 5′>gaagttctcacgaatgtc<3′
*POLG1*	SNP89870432	Exon5	c.1399G>A	A467T	89870432	rs113994095	Forward 5′>aagcagggcaaacacaag<3′; Rerverse 5′>tggaagttctcacgaatgtc<3′
*POLG1*	SNP89866735	Exon10	c.2165G>A	R722H	89866735	rs185645212	Forward 5′>cttaccatcgtgacactg<3′; Rerverse 5′>tgaacccaaactctttcc<3′
*POLG1*	SNP89861828	Exon18	c.3708G>T	Q1236H	89861828	rs2307441	Forward 5′>ctggagttaattggtatgtag<3′; Rerverse 5′>ttacgaattgaggctagtc<3′
*POLG1*	SNP89866657	Exon11	c.2243G>C	W748S	89866657	rs113994097	Forward 5′>attctgaggactaaacaaag<3′; Rerverse 5′>aggaccaaagtagtgaag<3′
*POLG1*	SNP89865073	Exon13	c.2492A>G	Y831C	89865073	rs41549716	Forward 5′>gttggagcaaggagaaag<3′; Rerverse 5′>gcctgaaatcacactctg<3′
*POLG1*	SNP89860013	Exon21	c.3689C>T	S1230F	89860013	rs3179578	Forward 5′>ctggagttaattggtatgtag<3′; Rerverse 5′>ttacgaattgaggctagtc<3′
*POLG1*	SNP89864114	Exon16	c.2864A>G	Y955C	89864114	rs113994099	Forward 5′>ccttgagtccagttagtg<3′; Rerverse 5′>gtgcccagaacatatttac<3′
*POLG1*	SNP89870180	Exon8	c.1548G>A	E516E	89870180	New report	Forward 5′>ctcctgtggtcatttatg<3′; Rerverse 5′>agaaggaactctcaataag<3′

Notes.

a Offsets for each SNP were calculated relative to the translation start site of the gene mRNA (*POLG1* according to GenBank accession number NM_002693.2). Mutation nomenclature was verified using the Mutalyzer program (www.lovd.nl/mutalyzer/1.0.1/).

§ The ID was taken directly from the public SNP database (www.ncbi.nlm.nih.gov/SNP).

### Sequence variation analysis

We aligned multiple sequences of *POLG1* from different species using the Clustal W program.

### RT-PCR for alternative splicing analysis

Splicing efficiency of targeted exons was monitored using standard RT-PCR methods to amplify the relevant portions of mRNA using primers in the flanking exons. Approximately 1 µg of total RNA was reverse-transcribed with random primers using the SuperScript III first-strand synthesis kit (Invitrogen). Then, a 0.1 volume of this reaction was used for PCR amplification for 35 cycles under the following conditions: denaturation at 94°C for 30 s, annealing at 60°C for 30 s, and elongation at 72°C for 30 s. Primers used to assess inclusion of Intron11_E12 (including c.2070-12T>A and c.2070-21T>C) splicing were 5-aagcactgtctcgaacaggg-3 (sense) and 5-catggtgatagctgggctgg-3 (antisense). Primers used to assess splicing at Intron11_E12 acceptor sites were as follows: 5-aagcactgtctcgaacaggg-3 (sense); 5-cactgcagctcgcaagttct-3 (antisense); Primers used to assess inclusion of Intron19_E20 (including c.3105-11T>C and c.3105-36A>G) splicing were 5-gggagttgaacctcccagtg-3 (sense) and 5-gcatgaggtgtaagtagtca-3 (antisense). Primers used to assess splicing at Intron11_E12 acceptor sites were as follows: 5-gggagttgaacctcccagtg-3 (sense); 5-ggggtacgtggtatgtcaga-3 (antisense).

### Isolation of peripheral blood lymphocytes from patients' blood samples

Peripheral blood lymphocytes were separated from other blood components using the Ficoll method (Amersham). The blood samples were diluted with sterile PBS 1∶1. The diluted blood then carefully poured onto the Ficoll solution. Harvest the ring with white blood cells without touching the Ficoll using a sterile pipette tips after centrifugation of the tubes 20 min at 1600 rpm. We diluted the peripheral blood lymphocytes with PBS and washed them twice in PBS. Peripheral blood lymphocytes were cultured in RPMI1640 medium supplemented with 10% (v/v) FBS (Invitrogen).

### Quantitative mRNA levels of *POLG1*

The mRNA levels of *POLG1* in human peripheral blood lymphocytes were measured with real-time PCR using gene-specific primers as follows: 5-aagcactgtctcgaacaggg-3 (sense); 5-cactgcagctcgcaagttct-3 (antisense); For real-time PCR, 1 µL of cDNA was amplified for 45 cycles with a master mix (SYBR Green Supermix; ABI) using a thermo cycler (MJ). Melting curve analysis was done at the end of the reaction (after 45 cycles) to assess the quality of the final PCR products. The threshold cycle C(t) values were calculated by fixing the basal fluorescence at 0.05 unit. Three replicates were used for each sample, and the average C(t) value was calculated. The ΔC(t) values were calculated as C(t) sample – C(t) *GAPDH* (or β-*Actin*). The N-fold increase or decrease in expression was calculated by the ΔΔCt method using the C(t) value as the reference point.

### MtDNA copy number quantification

The measure of mtDNA copy number was performed on peripheral blood lymphocytes by quantitative RT-PCR based on SYBR-Green I fluorescence. Similar to previous analysis [17], the amount of mtDNA was compared with the amount of the nuclear gene encoding the β-*globin* gene, contained in the same sample. The fluorescent signal intensities were recorded and analyzed during PCR in the thermo cycler. The mtDNA/β-*globin* gene DNA ratio obtained in the patient's samples was expressed as a percentage of the mean value obtained in control samples, which represent the 100% value.

### SDS-PAGE and Western blot analysis

Patients' blood cell lysates were mixed with equal volume of Laemmli buffer (62.5 mM Tris-HCl pH 6.8, 2% sodium dodecyl sulfate (SDS), 50 mM Dithiothreitol (DTT), 10% glycerol, 0.01% bromophenol blue), boiled for 3 min at 100°C. Electrophoresis of blood cell extracts from PD patients and health controls was done under denaturing reducing conditions using 4%–12% Nupage gels with Mops-SDS running buffer (Invitrogen). Approximately 25 µg of protein was used per lane for analysis as described in the protocol [18]. Electrophoresis was for approximately 60 minutes at 150 V. After electrophoresis, the proteins were transferred to nitrocellulose membranes (Amersham) using the Surelock X-cell transfer system (Invitrogen). Then the membrane was blocked in TBS/T buffer (1× TBS containing 0.05% Tween 20) with 5% BSA and probed with appropriate primary antibody: antibody against POLG1 (Abcam Catalog#Ab128899, 1∶2000 dilution) and Rabbit secondary antibodies (Abcam, 1∶2000 dilution) coupled to horseradish peroxidase. The blots were washed in TBS/T, incubated using the ECL kit (Cell Signaling Technology), and exposed to X-ray film (Kodak). GAPDH/PORIN levels were detected as loading controls by antibodies against GAPDH and PORIN (Abcam).

### Statistical analysis

Genetic association and the Hardy-Weinberg equilibrium for the distribution of genotypes were tested by χ^2^ analysis with Yates' correction. Odds ratios (ORs) were calculated with 95% confidence intervals (CI). The pairwise linkage disequilibrium (LD) coefficient r2 was calculated using the program Haploview from the Broad Institute website (http://www.broad.mit.edu/mpg/haploview/index.php) and German Research Center for Environmental Health's website (http://ihg2.helmholtz-muenchen.de/ihg/snps.html). P less than 0.05 was considered statistically significant. We defined pairs to be in strong LD if one side upper 95% confidence bound on D′ is >0.98 and lower bound is >0.7. Fraction of strong LD in informative comparisons must be at least 0.95.

## Results

### *POLG1* exonic and intronic SNP screening

**Figure 1:**
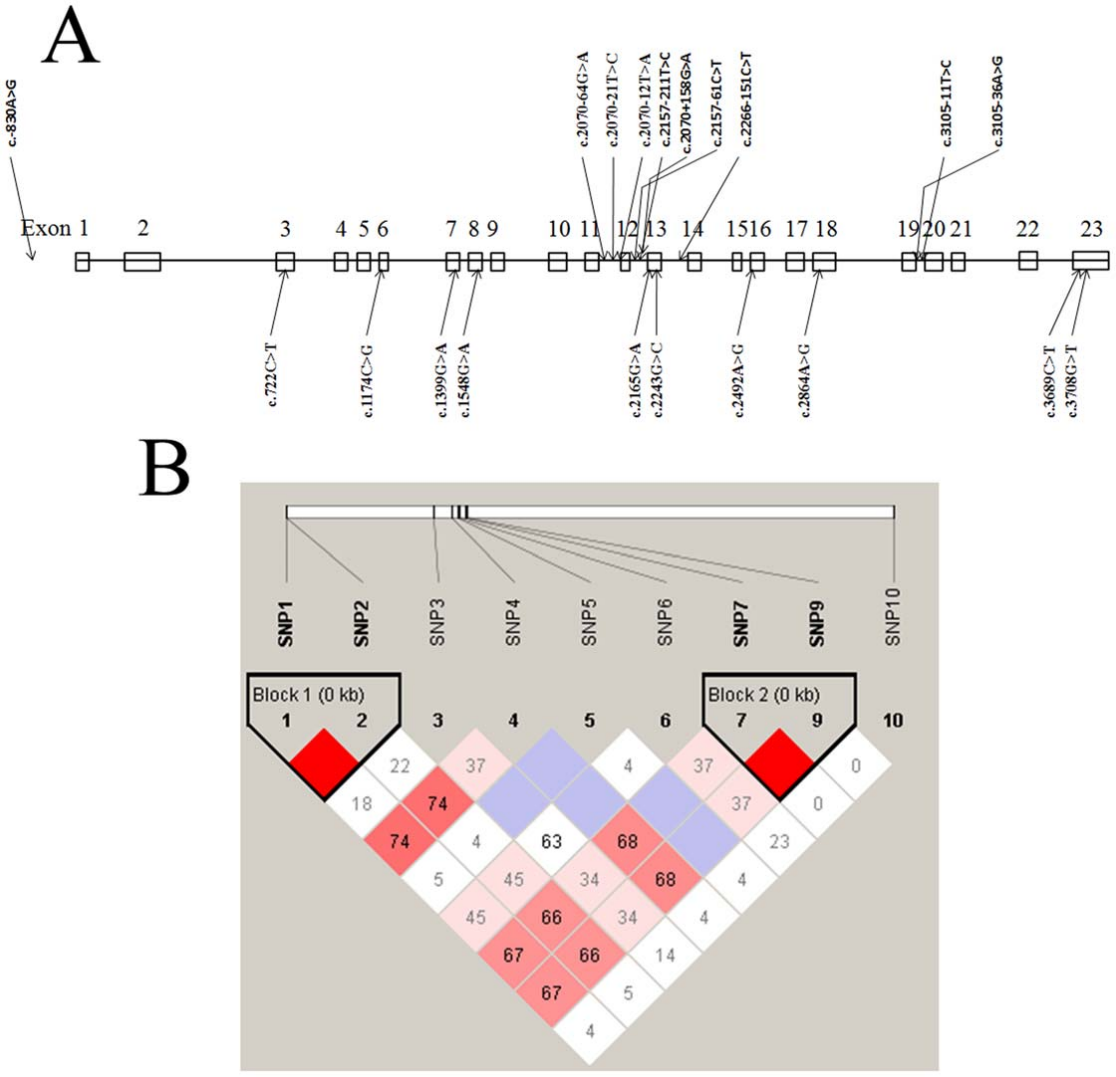
*POLG1* SNP position and linkage disequilibrium analysis. (A) Position of the *POLG1* gene exonic and intronic SNP variations. (B) Pair-wise linkage disequilibrium analysis in the *POLG1* gene for block prediction. Linkage disequilibrium (LD) coefficients (|D′| and r2) between nine intronic SNPs of *POLG1* gene are graphically indicated. Triangles indicate haplotype blocks.

By direct DNA sequencing in twenty-five individuals, we identified twenty SNPs within the *POLG1* gene, including ten exonic SNPs and ten intronic SNPs. Among twenty SNPs identified, seventeen SNPs were already registered in the dbSNP database, and three SNPs are novel (Table 1, Figure 1A). Twenty identified SNPs were selected for larger scale genotyping in 344 PD patients and 154 health controls for PD genetic association study based on frequencies and/or locations.

### Association of exonic and intronic SNPs with PD

Cases and controls were genotyped for ten exonic SNPs including the five coding region mis-sense substitutions previously investigated (rs56410699: c.722C>T-P241L; rs145289229: c1174C>G-L392V; rs185645212: c.2165G>A-R722H; rs41549716: c.2492A>G-Y831C; and rs3179578: c.3689T>C-S1230F), and four alleles previously described in the UK population (rs113994095: c.1399G>A-A467T; rs113994097: c.2243G>C-W748S; rs113994099: c.2864A>G-Y955C; and rs2307441: c.3708G>T-Q1236H), and novel reported c.1830G>A-E516E. All of the exonic variant alleles were in Hardy Weinberg equilibrium. We did not identify any significant association between these ten exonic SNPs and PD (P>0.1, Table 2). To determine whether the alleles were acting in combination, we compared compound genotypes in PD cases and controls. There was no difference in the frequency of compound genotypes defined by L392V, W748S, Y831C, S1230F, and Q1236H (data not shown).

**Table 2:** Analysis of association of ten *POLG1* coding polymorphisms with PD.

				Genotype frequency				Allele	Frequency	
SNP^1^	Location	N^2^		Genotype		p-value^3^	OR(95%CI)^4^	Allele	p-value^5^	OR(95%CI)^4^
	Exon8		GG	G/A	AA			A		
c.1548G>A,E516E	cases	343	0.500	0.308	0.192	0.678	0.925(0.632–1.353)	0.346	0.218	0.840(0.636–1.109)
	controls	154	0.480	0.266	0.254			0.387		
	Exon2		CC	C/T	TT			T		
c.722C>T,P241L	cases	344	0.819	0.107	0.074	0.371	0.806(0.502–1.294)	0.127	0.326	0.824(0.561–1.212)
	controls	154	0.785	0.129	0.086			0.150		
	Exon4		CC	C/G	GG			G		
c.1174C>G,L392W	Cases	344	0.270	0.488	0.242	0.514	1.150(0.756–1.748)	0.486	0.199	1.193(0.911–1.563)
	controls	154	0.298	0.519	0.183			0.442		
	Exon5		GG	G/A	AA			A		
c.1399G>A,A467T	Cases	344	0.848	0.110	0.042	0.057	0.629(0.388–1.017)	0.097	0.028	0.637(0.423–1.001)
	controls	154	0.779	0.155	0.066			0.138		
	Exon10		GG	G/A	AA			A		
c.2165G>A,R722H	Cases	344	0.377	0.459	0.164	0.253	0.791(0.530–1.183)	0.393	0.033	0.745(0.568–0.978)
	controls	154	0.324	0.422	0.254			0.465		
	Exon18		GG	G/T	TT			T		
c.3708G>T,Q1236H	Cases	344	0.427	0.319	0.254	0.666	1.088(0.742–1.595)	0.413	0.281	1.164(0.883–1.533)
	controls	154	0.448	0.350	0.202			0.377		
	Exon11		GG	G/C	CC			C		
c.2243G>C,W748S	Cases	344	0.828	0.139	0.033	0.171	1.471(0.843–2.565)	0.102	0.391	1.229(0.767–1.969)
	controls	154	0.876	0.077	0.047			0.085		
	Exon13		AA	A/G	GG			G		
c.2492A>G,Y831C	Cases	344	0.918	0.058	0.024	0.296	0.714(0.378–1.348)	0.053	0.034	0.575(0.342–0.965)
	controls	154	0.889	0.045	0.064			0.086		
	Exon21		CC	C/T	TT			T		
c.3689C>T,S1230F	Cases	344	0.729	0.209	0.062	0.514	0.870(0.572–1.323)	0.166	0.453	0.875(0.616–1.242)
	controls	154	0.701	0.227	0.072			0.186		
	Exon16		AA	A/G	GG			G		
c.2864A>G,Y955C	Cases	344	0.627	0.250	0.123	0.856	1.037(0.699–1.539)	0.248	0.816	1.038(0.750–1.420)
	controls	154	0.636	0.246	0.118			0.241		

1 SNP name for genotype in cases and controls.

2 Number of valid subjects who were successfully genotyped for each of SNP.

3 Analysis performed by a 2×2 table for each SNP using major homozygotes vs. others in cases and controls.

4 Reference group (controls) designated with an OR of 1.00.

5 Analysis performed by a 2×2 table for the number of each allele in cases and controls.

**Figure 2:**
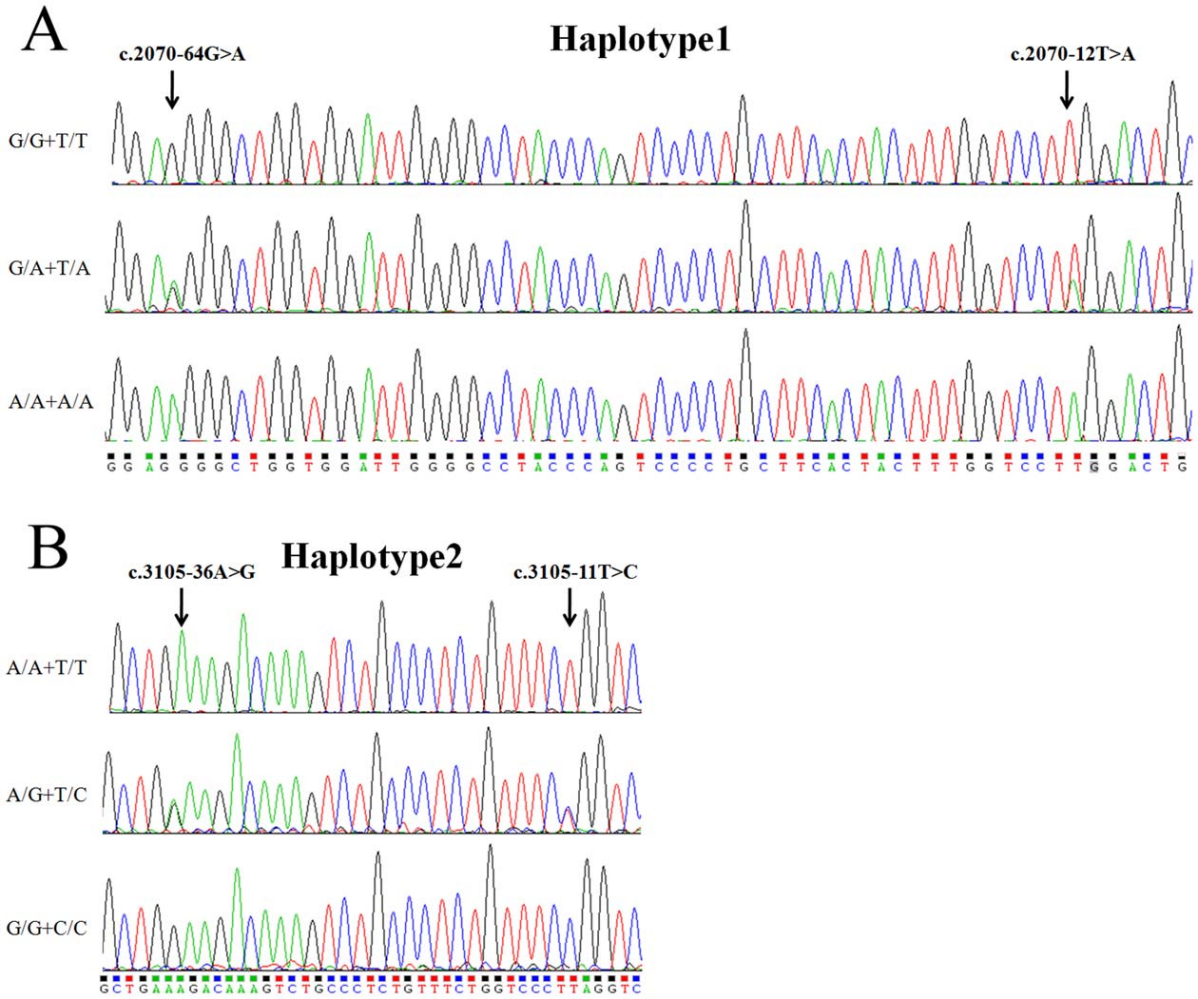
Genomic DNA sequence electropherograms of the two intronic haplotype of *POLG1* gene are shown. Panel A shows c.2070-12T>A and c.2070-64G>A in intron 11 of *POLG1* gene; Panel B shows c.3105-11T>C and c.3105-36A>G in intron 19 of *POLG1* gene.

However, four intronic SNPs were significantly associated with PD among ten intronic SNPs of the *POLG1* gene (Table 3). The statistical P-value of new reported SNP: c.2070-12T>A and rs2307439: c.2070-64G>A in intron 11 was 0.00011 (OR = 1.727) for absolute allele frequencies. C.2070-12T>A and c.2070-64G>A were in absolute linkage disequilibrium (Haplotype 1, ht1; Figure 2A). Furthermore, a comparison of allelic frequencies in the haplotype 1 indicated that ht1-AA was significantly more frequent in PD individuals than in the healthy control subjects (P = 0.00011). Another pair of polymorphisms between rs2302084: c.3105-11T>C and rs2246900: c.3105-36A>G in intron 19 was detected as significant association with PD (Haplotype 2 (ht2); P = 0.00031, OR = 1.648) (Figure 2B). To investigate the pattern of LD in the *POLG1* locus, pairwise LD was measured by D′ values among nine intronic polymorphisms (Figure 1B). Linkage disequilibrium analysis indicated that c.2070-12T>A and c.2070-64G>A in ht1 as well as c.3105-11T>C and c.3105-36A>G in ht2 could be parsed into two different blocks, which had a strong LD spine. Furthermore, we confirmed our association analysis between the two intronic haplotypes and PD in an independent case-control sample consisting of 110 individuals with PD and 130 normal controls from the Hubei province of China. The statistical P-value of c.2070-12T>A and c.2070-64G>A in intron 11 was 0.00058 (OR = 1.896); similarly the statistical P-value of c.3105-11T>C and c.3105-36A>G in intron 19 was 0.0063 (OR = 1.654) (Table 4). These results demonstrated that intronic SNPs rather than exonic SNPs of *POLG1* were associated with PD development in Chinese Parkinsonism populations.

**Table 3:** Analysis of association of ten *POLG1* intronic polymorphisms with PD.

				Genotype frequency				Allele	frequency	
SNP^1^	Location	N^2^		Genotype		p-value^3^	OR(95%CI)^4^	Allele	p-value^5^	OR(95%CI)^4^
	Intron11		GG	G/A	AA			A		
c.2070-64G>A	cases	341	0.279	0.477	0.244	0.014	1.649(1.105–2.461)	0.482	0.00011	1.727(1.308–2.280)
	controls	154	0.389	0.519	0.091			0.350		
	Intron11		TT	T/C	CC			C		
c.2070-21T>C	cases	344	0.291	0.436	0.273	0.311	0.799(0.518–1.234)	0.491	0.950	0.990(0.758–1.297)
	controls	154	0.246	0.519	0.235			0.495		
	Intron11		TT	T/A	AA			A		
c.2070-12T>A	Cases	344	0.279	0.477	0.244	0.014	1.649(1.105–2.461)	0.463	0.00011	1.727(1.308–2.280)
	Controls	154	0.389	0.519	0.091			0.350		
	Intron12		TT	T/C	CC			C		
c.2157-211T>C	Cases	342	0.915	0.066	0.019	0.457	1.326(0.629–2.793)	0.052	0.292	1.447(0.725–2.889)
	Controls	154	0.935	0.062	0.003			0.034		
	Intron12		CC	C/T	TT			T		
c.2157-61C>T	Cases	344	0.386	0.465	0.149	0.235	0.786(0.527–1.171)	0.381	0.022	0.728(0.555–0.956)
	controls	154	0.331	0.422	0.247			0.458		
	Intron13		CC	C/T	TT			T		
c.2266-151C>T	Cases	343	0.337	0.468	0.195	0.566	1.123(0.755–1.671)	0.429	0.561	1.084(0.825–1.424)
	controls	154	0.363	0.454	0.183			0.410		
	Intron19		AA	A/G	GG			G		
c.3105-36A>G	Cases	344	0.232	0.482	0.285	0.014	1.682(1.108–2.553)	0.526	0.00031	1.648(1.255–2.164)
	controls	154	0.337	0.519	0.142			0.401		
	Intron19		TT	T/C	CC			C		
c.3105-11T>C	Cases	344	0.232	0.482	0.285	0.014	1.682(1.108–2.553)	0.526	0.00031	1.648(1.255–2.164)
	controls	154	0.337	0.519	0.142			0.401		
	Intron10		GG	G/A	AA			A		
c.2070+158G>A	Cases	344	0.741	0.209	0.050	0.897	1.029(0.665–1.592)	0.154	0.942	0.987(0.681–1.430)
	controls	154	0.746	0.194	0.060			0.157		
	promoter		AA	A/G	GG			G		
c.-830A>G	Cases	344	0.627	0.250	0.126	0.517	0.879(0.596–1.298)	0.251	0.520	0.905(0.666–1.222)
	controls	154	0.597	0.272	0.131			0.267		

1 SNP name for genotype in cases and controls.

2 Number of valid subjects who were successfully genotyped for each of SNP.

3 Analysis performed by a 2×2 table for each SNP using major homozygotes vs. others in cases and controls.

4 Reference group (controls) designated with an OR of 1.00.

5 Analysis performed by a 2×2 table for the number of each allele in cases and controls.

**Table 4:** Analysis of association of *POLG1* intronic haplotypes with PD in an independent case-control sample.

				Genotype frequency				Allele	frequency	
SNP^1^	Location	N^2^		Genotype		p-value^3^	OR(95%CI)^4^	Allele	p-value^5^	OR(95%CI)^4^
	Intron11		GG	G/A	AA			A		
c.2070-64G>A	cases	110	0.245	0.482	0.273	0.021	1.921(1.098–3.363)	0.499	0.00058	1.896(1.315–2.735)
	controls	130	0.384	0.515	0.101			0.358		
	Intron11		TT	T/A	AA			A		
c.2070-12T>A	Cases	110	0.245	0.482	0.273	0.021	1.921(1.098–3.363)	0.499	0.00058	1.896(1.315–2.735)
	Controls	130	0.384	0.515	0.101			0.358		
	Intron19		AA	A/G	GG			G		
c.3105-36A>G	Cases	110	0.227	0.472	0.301	0.076	1.680(0.944–2.991)	0.537	0.0063	1.654(1.152–2.376)
	controls	130	0.330	0.515	0.155			0.412		
	Intron19		TT	T/C	CC			C		
c.3105-11T>C	Cases	110	0.227	0.472	0.301	0.076	1.680(0.944–2.991)	0.537	0.0063	1.654(1.152–2.376)
	controls	130	0.330	0.515	0.155			0.412		

1 SNP name for genotype in cases and controls.

2 Number of valid subjects who were successfully genotyped for each of SNP.

3 Analysis performed by a 2×2 table for each SNP using major homozygotes vs. others in cases and controls.

4 Reference group (controls) designated with an OR of 1.00.

5 Analysis performed by a 2×2 table for the number of each allele in cases and controls.

**Figure 3:**
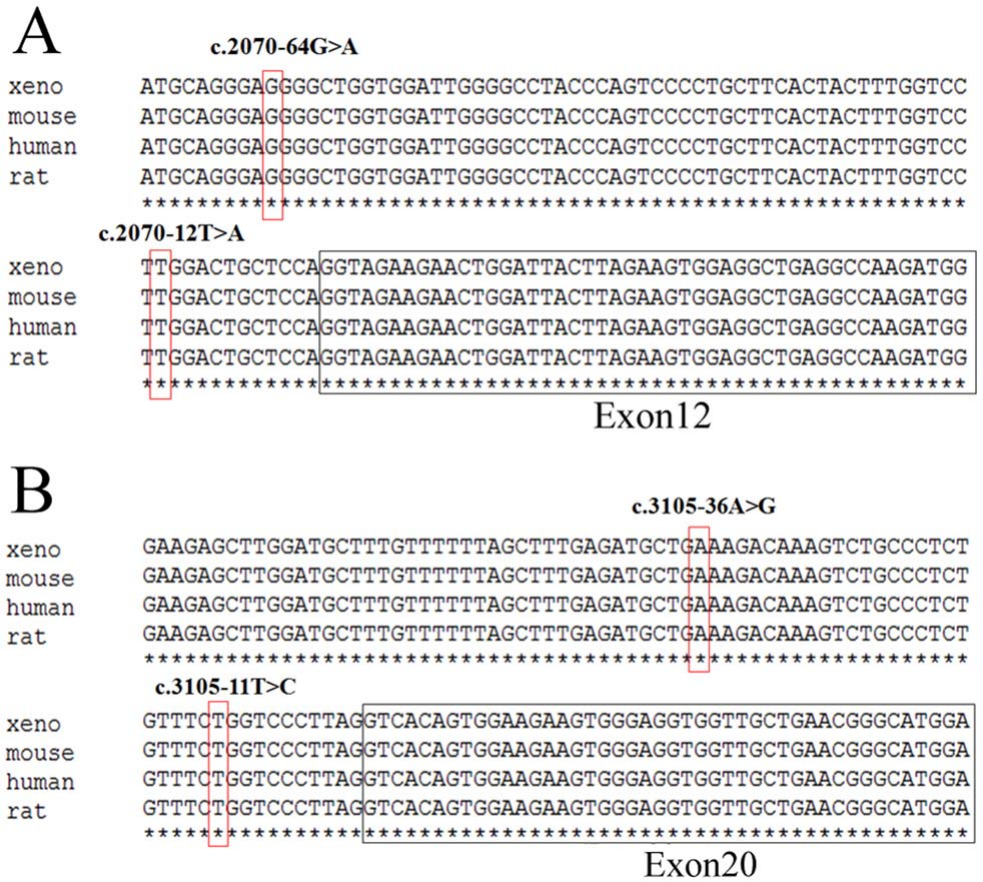
*POLG1* sequence alignment and intronic polymorphisms are evolutionarily conversed. Multiple sequence alignments of *POLG1* homologous sequences in different species (Human, Mouse, Rat, Xenopus laevis) are shown. DNA variations reported in this study are highlighted in red rectangle. Panel A shows c.2070-12T>A and c.2070-64G>A in intron 11 of *POLG1* gene; Panel B shows c.3105-11T>C and c.3105-36A>G in intron 19 of *POLG1* gene.

Furthermore, c.2070-12T>A and c.2070-64G>A substitution in intron 11 as well as c.3105-11T>C and c.3105-36A>G in intron 19 occur highly conserved (Figure 3A and 3B). These intronic substitutions just happened to be nearby the pyrimidine-rich region of the 3′ splicing acceptor. Mutations within the pyrimidine-rich intronic sequence could cause human diseases [19], [20], [21]. Therefore, it is of great interest and importance to examine the effect of these four polymorphisms on *POLG1* RNA splicing.

### Association of *POLG1* poly-Q alleles with PD

In order to study whether the possible effect of length alleles of the *POLG1* poly-Q tract in 498 Chinese PD patients vs. controls, we amplified *POLG1* exon 2 and performed sequencing on an ABI 3100 automatic sequencer. We found variations of the poly-Q tract ranging from 6Q up to 14Q in our recruited materials (Table 5). The genotype distributions for the two most common alleles (10Q and 11Q) did not deviate from Hardy-Weinberg equilibrium in PD patients or healthy controls. For statistical analysis the alleles were divided into two main groups; 10/11Q variants (10 and 11 repeats) and non-10/11Q variants (6, 7, 8, 9, 12, 13 and 14 repeats). We found a significant association (P = 0.03, OR = 2.16) of the non-10/11Q variants with PD (Table 6). These results indicated that non-10Q/11Q viriants of *POLG1* gene other than the conserved 10/11Q allele might increase susceptibility to PD in Chinese populations.

**Table 5:** *POLG1* exon 2 poly-Q allele frequencies in our study.

Poly-Q	PD patients (n = 344)	PD patients (n = 344)	Healthy controls (n = 154)	Healthy controls (n = 154)
	2n	%	2n	%
6Q	0	0	1	0.6
7Q	3	0.8	1	0.6
8Q	2	0.6	2	1.3
9Q	21	6.1	2	1.3
10Q	275	80	132	85.7
11Q	24	6.9	12	7.7
12Q	14	4.0	2	1.3
13Q	4	1.1	2	1.3
14Q	1	0.3	0	0
Σ	344	100	154	100

**Table 6:** Comparison of *POLG1* gene Q10 and Q11 vs. non-Q10 and non-Q11 poly-Q allele frequencies in patients with PD patients and healthy controls.

Alleles	PD patients (n = 344)	PD patients (n = 344)	Healthy Control (n = 154)	Healthy Control (n = 154)
	2n	%	2n	%
10/11Q (%)	299	86.9	144	93.5
Non-10/11Q (%)	45	13.1	10	6.5
Fisher's exact test	P = 0.03			
Odds ratio	2.16			
95% CI	1.06–4.42			

### Intronic SNPs associated with PD don't influence *POLG1* mRNA alternative splicing

Substantial evidences indicated that misregulation of alternative splicing was associated with human diseases, including PD [22], [23]. To explore the molecular mechanism of association between intronic SNP variants of *POLG1* and PD, the ability of the intron to influence pre-mRNA splicing was tested.

**Figure 4:**
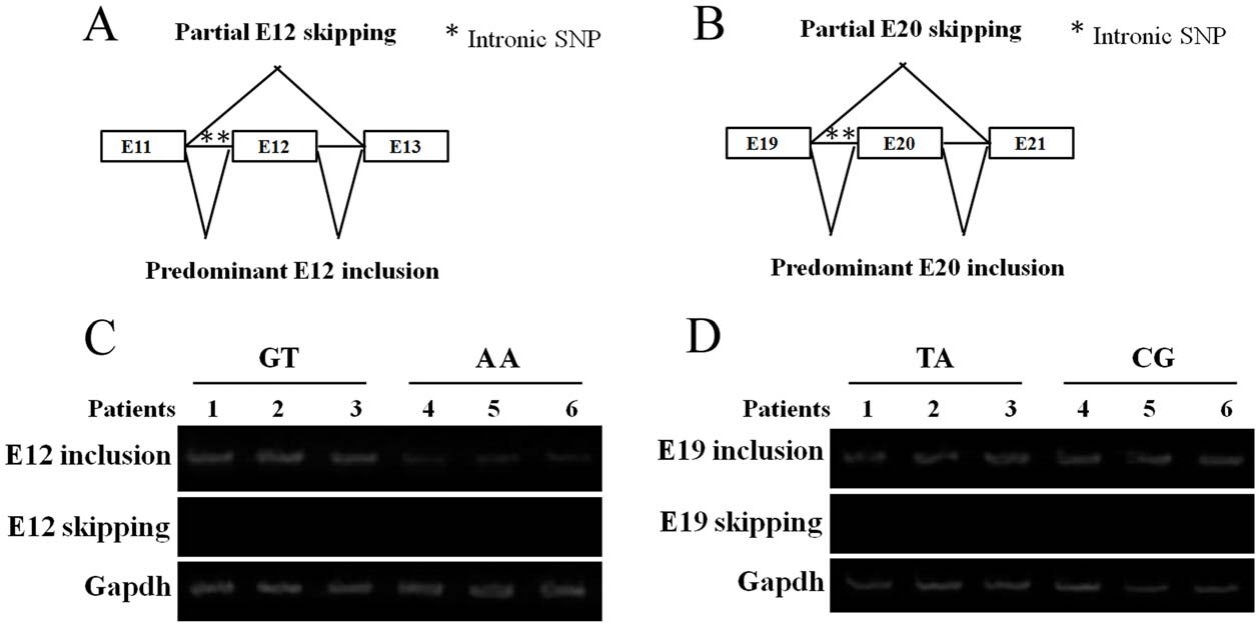
*POLG1* intronic SNPs don't influence *POLG1* mRNA alternative splicing. Diagram A and B shows the normal splicing patterns in which *POLG1* E12 (A) and E19 (B) are predominantly included in human and the partially exon skipping that occurs in E12 and E19 are alternatively spliced. Panel C and D shows RT-PCR analysis of *POLG1* E12 (C) and E19 (D) splicing patterns in patients' blood (n = 3).

C.2070-12T>A and c.2070-64G>A in the intron 11 were located in the 64 bp upstream of exon 12, and haplotype 2 (c.3105-11T>C and c.3105-36A>G in intron 19) was located in the 36 bp upstream of exon 20. Both haplotype SNPs are within several serine/arginine-rich protein-binding motifs (Figure 4A and 4B). Among these, a predicted SF2/ASF-binding site implicated in splicing scored highest (ESE finder), which was deleted by the T allele instead of the C allele (CAGCGTA to CAGTGTA) of haplotype 1 in intron 11 of *POLG1*. However, RT-PCR amplification of transcripts from exon 11 to exon 12 as well as exon 19 to exon 20 of *POLG1* did not reveal any splice variants, regardless of C or T alleles (Figure 4C and 4D). This result argues against altered splicing as primary mechanism, because if intron 11 and intron 19 were to affect pre-mRNA splicing, we would expect to see different allelic RNA ratios in the blood samples of these patients carrying haplotype 1 of c.2070-12T>A and c.2070-64G>A as well as haplotype 2 of c.3105-11T>C and c.3105-36A>G. Therefore, intronic polymorphism variants associated with PD couldn't influence *POLG1* mRNA splicing. However, because of lack of primary brain tissues from the patients, we could not further examine the effect of intronic SNPs associated with PD on RNA splicing.

### Intronic SNPs associated with PD affect *POLG1* mRNA and protein levels

**Figure 5:**
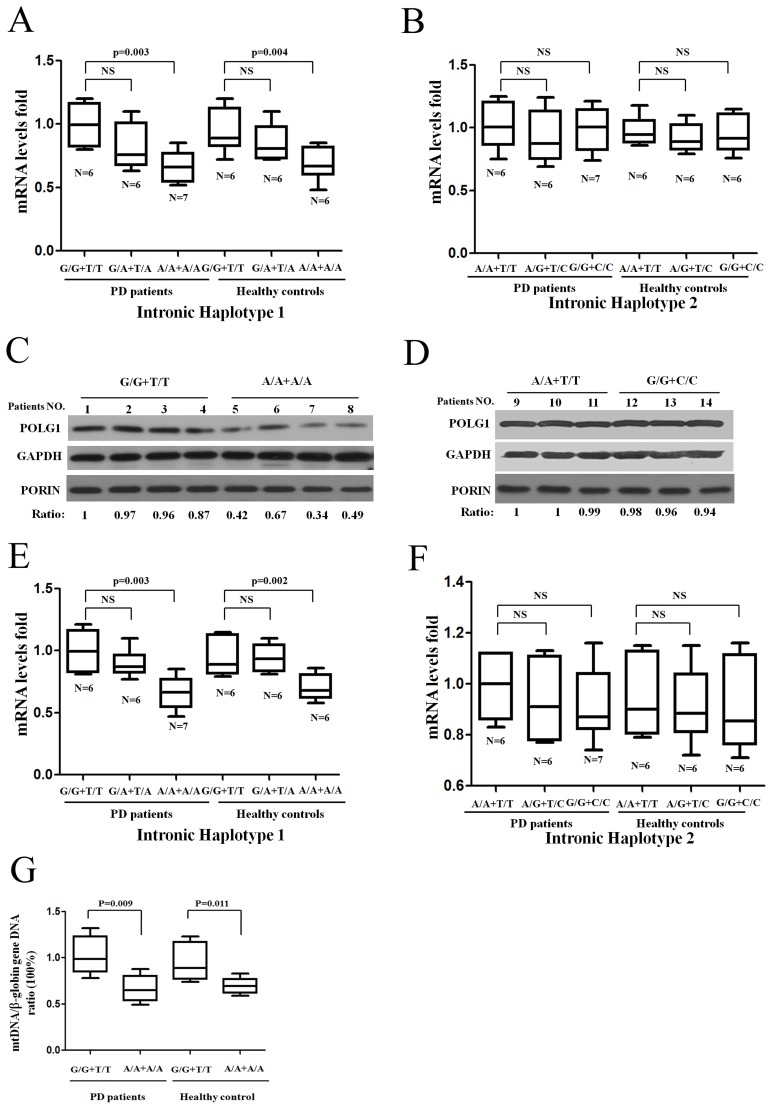
Intronic SNPs of *POLG1* gene associate with decreased *POLG1* mRNA and protein levels. (A) Quantitative RT-PCR of *POLG1* mRNA from patients' peripheral blood lymphocytes with the main G/G+T/T, A/A+A/A genotypes and heterogeneous G/A+T/A genotype carriers of haplotype 1. (B) Quantitative RT-PCR of *POLG1* mRNA shown there was no effect of haplotype 2 genotypes on *POLG1* mRNA transcription. Panel C and D shows western blot of POLG1 protein levels from eight age and gender-matched patients carrying haplotype 1 genotypes (G/G+T/T, A/A+A/A) as well as six patients carrying haplotype 2 genotypes (A/A+T/T, G/G+C/C). PORIN protein was detected as a mitochondrial loading control. The signal ratio was obtained through comparing POLG1 protein band intensity with GAPDH protein intensity. (E and F) *POLG1* mRNA was detected by quantitative RT-PCR from isolated and cultured patients' peripheral blood lymphocytes from different individuals' carrying with different genotypes of haplotype 1 or haplotype 2. (G) Intronic polymorphisms effect of *POLG1* haplotype 1 and haplotype 2 on mtDNA copy number.

In addition to alternative splicing, mutations within the pyrimidine-rich intronic sequence could also affect mRNA transcription [22], [23]. Although *POLG1* expression varies widely among individuals, the contribution of genetic factors remains uncertain. Here we isolated 74 peripheral blood lymphocytes from blood samples of PD patients (six PD patients or healthy controls carrying three different genotypes of haplotype 1 or haplotype 2) to measure *POLG1* mRNA levels by quantitative RT-PCR. *POLG1* mRNA levels from six PD patients' peripheral blood lymphocytes with the main G/G+T/T genotype of haplotype1 in intron 11 had 1.7 fold (95%CI: 1.1–2.8) higher levels than A/A+A/A carriers combined (P = 0.003 in PD patients and P = 0.004 in healthy controls), with no interactions between genotypes and sex (Figure 5A). To further normalize the *POLG1* mRNA levels we observed, another endogenous reference control, β-*Actin*, was used to detect similar decreasing effect on *POLG1* mRNA expression in haplotype 1-A/A+A/A genotype carriers (Figure S1A and S1B). After adjusting for sex ratio in each genotype, intronic c.2070-12T>A and c.2070-64G>A polymorphisms in intron 11 remained significantly associated with decreased *POLG1* expression (1.67-fold G/G+T/T over G/A+T/A and A/A+A/A, 95%CI: 1.11-2.46, P<0.014) (data not shown). Decreased POLG1 levels in whole blood protein levels was also detected using 4 G/G+T/T carriers and 4 PD patients carrying A/A+A/A genotype in haplotype 1 (Figure 5C). We also detected the effect of haplotype 2 (c.3105-11T>C and c.3105-36A>G) in intron 19 on *POLG1* mRNA transcription in peripheral blood lymphocytes and protein levels in whole blood cells, and found there was no significant association between *POLG1* mRNA levels, protein levels and different genotypes of haplotype 2 (Figure 5B and 5D). Furthermore, isolated peripheral blood lymphocytes were cultured for three days in RPMI1640 medium supplemented with 10% FBS. We found a similar decrease of *POLG1* mRNA levels in haplotype 1 A/A+A/A in intron 11 of the *POLG1* gene (P = 0.003 in PD patients; and P = 0.002 in healthy controls, Figure 5E and F). There were no other mutations of *POLG1* gene found and no intronic region methylation in the same region found by sequencing and bisulfate-sequencing PCR (unpublished data). We concluded that intronic c.2070-12T>A and c.2070-64G>A in the intron 11 were associated with decreased *POLG1* mRNA level and protein levels in human blood lymphocytes. However, *POLG1* expression maybe differently regulated in different human tissues such as blood and brain tissue, differential *POLG1* levels due to intronic polyporphisms still need to be confirmed by primary brain tissues. Given the significant reduction in *POLG1* expression in ht1-A/A+A/A carriers, we further investigated the potential effect on mtDNA copy number. The results of qRT-PCR showed a reduction of the mtDNA/β-globin gene DNA ratio of 69%±8% (P = 0.009 in ten PD patients carrying ht1-A/A+A/A and ht1-G/G+T/T) and 73%±8% (P = 0.011 in ten healthy controls carrying ht1-A/A+A/A and ht1-G/G+T/T) in ht1-A/A+A/A carriers compared with ht1-G/G+T/T carriers in both PD patients and age-matched control individuals respectively (Figure 5G). These data indicated that c.2070-12T>A and c.2070-64G>A in the intron 11 changed mtDNA replication.

## Discussion

Since *POLG1* is essential for mtDNA stability, dysfunction of which has been demonstrated in PD patients, it is a good candidate for PD susceptibility gene. Numerous previous association studies between *POLG1* polymorphisms and PD were reported in several ethnic groups, but the results were inconsistent. Parkinsonism-associated *POLG1* variant Y831C was found to be associated with PD in Finnish populations [14]. Recently two genome-wide association studies (GWAS) performed in a North American, UK [24] and German material of Caucasian origin with sporadic PD and North American material of Caucasian origin with familial PD [25], screened for a missense polymorphism rs3087374 (Q1236H) in *POLG1*, but no significant association with PD was found. In this study, we identified two *POLG1* intronic haplotypes containing two neighboring SNPs significantly associated with Parkinsonism in Chinese populations. To our knowledge, this is the first report regarding association between intronic haplotype SNPs of *POLG1* and PD.

We examined twenty *POLG1* SNPs on the association with PD and found that four polymorphisms (c.2070-12T>A and c.2070-64G>A in intron 11; c.3105-11T>C and c.3105-36A>G in intron 19) were significantly associated with PD. The association between haplotype 1,2 and PD was further confirmed in an independent case-control samples consisting of 110 individuals with PD and 130 normal controls from the Hubei province of China. However, there was no significant association between PD and ten exonic SNPs. These results were consistant with previous report [26]. Therefore, no additional SNP was detected in other introns of the gene that could account for the observed associations in the population, indicating that disease risk was not associated with SNPs in other parts of *POLG1* gene, such as the 5′ promoter region, the 3′UTR, or the other exons. Furthermore, we found a significant association (P = 0.03, OR = 2.16) of the non-10/11Q variants with PD from a further case and control study on association of *POLG1* poly-Q alleles. Our results supported previous finding that *POLG1* poly-Q alleles other than the conserved 10/11Q allele may increase susceptibility to PD in Chinese populations [6], [15]. These results indicated that *POLG1* polymorphisms may exert a substantial influence on the inheritance of Parkinsonism in Chinese populations.

Although positive associations of exonic polymorphisms or mutation screening of *POLG1* with PD were reported, we failed to observe any association with susceptibility to PD for exonic polymorphisms of *POLG1*. Several factors could provide plausible explanations, e.g., (1) possible genetic differences between different ethnic groups; (2) Given the limited size of our study cohort, especially normal controls (n = 154), we are not able to exclude the possibility that rare *POLG1* alleles are associated with a small increased risk of the disorder; (3) different study designs and possible biases in recruiting case and controls among studies; (4) and the statistical approach in the previous study did not account for multiple significance testings, raising the possibility of a false positive result. Taking all together, our observations indicated that POLG1 amino acid substitutions were of course recessive and did not cause PD, but cause other severe neurological phenotypes.

Exonic splicing enhancers (ESEs), intronic splicing enhancers (ISEs), exonic splicing silencers (ESSs), and intronic splicing silencers (ISSs) were the known cis-acting sequence elements for promoting splice-site recognition [22], [27], [28]. These sequences tend to be short (typically ∼5–10 nt in length) and consist of relatively degenerate consensus sequences that are recognized by trans-acting splicing factors, such as serine/arginine-rich domain proteins (SR proteins) and nuclear ribonucleoprotein proteins (hnRNPs), which can regulate the use of a splice site [23], [29]. Intronic SNPs in the intron-exon boundaries were reported to have consequences for transcription factor binding sites and native splicing sites [27], [28], [30]. We initially studied possible alternative splicing of *POLG1* mRNA. Semiquantitative RT-PCR indicated that endogenous E12 and E19 were not skipped by alternative splicing in blood from patients carrying c.2070-12T>A and c.2070-64G>A substitution in intron 11, or c.3105-11T>C and c.3105-36A>G in intron 19. Further study indicated that intronic c.2070-12T>A and c.2070-64G>A of *POLG1* associated with decreased its mRNA level and protein levels. However, mechanistic studies with minigenes of *POLG1* in transfected cells will be needed to discover multifactorial regulation of alternative splicing, including identifying positive and negative regulatory motifs in exon 12, exon 19, and in flanking introns. There are many reasons for intronic variants regulating mRNA transcription. The SF2/ASF protein was reported to have additional functions beyond splicing, such as regulating translation and stabilizing mRNA [31]. To test whether SF2/ASF was involved in intronic SNPs regulated *POLG1* mRNA, the expression of SF2/ASF mRNA in patients' blood was measured using real-time PCR, to account for tissue-specific effects of intronic SNPs on *POLG1* expression. However, similar SF2/ASF expression levels were detected in PD patients and health controls carrying c.2070-12T>A and c.2070-64G>A, or c.3105-11T>C and c.3105-36A>G of *POLG1*, respectively (data not shown). Further study will be needed for the intronic regions containing regulatory elements, like enhancer/attenuator elements that regulate transcription.

In summary, we examined the genetic association of *POLG1* with the risk of PD, and found the association of intronic haplotype variants of *POLG1* significantly linked to PD in Chinese populations. The results of this study could be helpful in understanding the important function of *POLG1* in PD development and in developing drugs to treat PD.

## Supporting Information

Figure S1 **Another endogenous reference control, β-** ***Actin*** **, is used to detect changes in** ***POLG1*** **expression.** (A) Quantitative RT-PCR of *POLG1* mRNA from patients' peripheral blood lymphocytes with the main G/G+T/T, A/A+A/A genotypes and heterogeneous G/A+T/A genotype carriers of haplotype 1. (B) Quantitative RT-PCR of *POLG1* gene shown there is no effect of haplotype 2 genotypes on *POLG1* mRNA transcription.
(TIF)
