# Supplementary figures and images for: Association of Mitochondrial DNA Polymerase γ Gene POLG1 Polymorphisms with Parkinsonism in Chinese Populations
Source: PLoS One. 2012 Dec 10;7(12):e50086. doi: 10.1371/journal.pone.0050086 (PMC3519471; doi:10.1371/journal.pone.0050086)

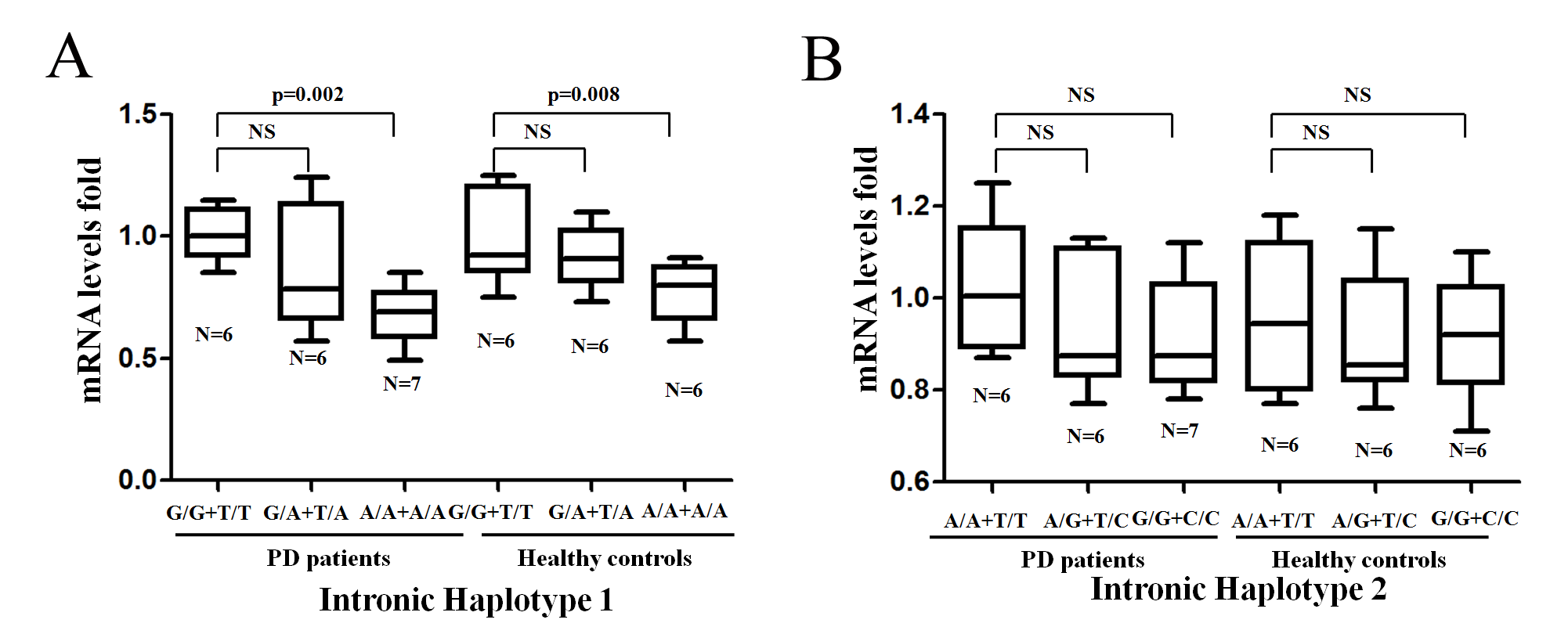

Supplement: Figure S1 — Another endogenous reference control, β- Actin , is used to detect changes in POLG1 expression. (A) Quantitative RT-PCR of POLG1 mRNA from patients' peripheral blood lymphocytes with the main G/G+T/T, A/A+A/A genotypes and heterogeneous G/A+T/A genotype carriers of haplotype 1. (B) Quantitative RT-PCR of POLG1 gene shown there is no effect of haplotype 2 genotypes on POLG1 mRNA transcription. (TIF) [file pone.0050086.s001.tif]
